# Molecular Typing of *Legionella pneumophila* Isolates in the Province of Quebec from 2005 to 2015

**DOI:** 10.1371/journal.pone.0163818

**Published:** 2016-10-05

**Authors:** Simon Lévesque, Cindy Lalancette, Kathryn Bernard, Ana Luisa Pacheco, Réjean Dion, Jean Longtin, Cécile Tremblay

**Affiliations:** 1 Laboratoire de santé publique du Québec, Institut national de santé publique du Québec, Sainte-Anne-de-Bellevue, Québec, Canada; 2 Département de microbiologie, infectiologie et immunologie, Université de Montréal, Québec, Canada; 3 Centre de recherche du centre hospitalier de l’Université de Montréal, Québec, Canada; 4 Public Health Agency of Canada, National Microbiology Laboratory, Winnipeg, Manitoba, Canada; 5 Département de médecine sociale et préventive, École de santé publique de l’Université de Montréal, Québec, Canada; 6 Centre de recherche en infectiologie de l’Université Laval, Québec, Canada; St Petersburg Pasteur Institute, RUSSIAN FEDERATION

## Abstract

*Legionella* is found in natural and man-made aquatic environments, such as cooling towers and hot water plumbing infrastructures. *Legionella pneumophila* serogroup 1 (*Lp*1) is the most common etiological agent causing waterborne disease in the United States and Canada. This study reports the molecular characterization of *Lp* strains during a 10 year period. We conducted sequence-based typing (SBT) analysis on a large set of *Lp* isolates (n = 284) to investigate the province of Quebec sequence types (STs) distribution in order to identify dominant clusters. From 2005 to 2015, 181 clinical *Lp* isolates were typed by SBT (141 sporadic cases and 40 outbreak related cases). From the same period of time, 103 environmental isolates were also typed. Amongst the 108 sporadic cases of *Lp*1 typed, ST-62 was the most frequent (16.6%), followed by ST-213 (10.2%), ST-1 (8.3%) and ST-37 (8.3%). Amongst other serogroups (SG), ST-1327 (SG5) (27.3%) and ST-378 (SG10) (12.2%) were the most frequent. From the environmental isolates, ST-1 represent the more frequent SBT type (26.5%). Unweighted pair group method with arithmetic mean (UPGMA) dendrogram from the 108 sporadic cases of SG1 contains 4 major clusters (A to D) of related STs. Cluster B contains the majority of the strains (n = 61) and the three most frequent STs in our database (ST-62, ST-213 and ST-1). During the study period, we observed an important increase in the incidence rate in Quebec. All the community associated outbreaks, potentially or confirmed to be associated with a cooling tower were caused by *Lp*1 strains, by opposition to hospital associated outbreaks that were caused by serogroups of *Lp* other than SG1. The recent major Quebec City outbreak caused by ST-62, and the fact that this genotype is the most common in the province supports whole genome sequencing characterization of this particular sequence type in order to understand its evolution and associated virulence factors.

## Introduction

Legionellosis has emerged in the second half of the 20^th^ century, and is caused by *Legionella pneumophila* (*Lp*) and related bacteria. Legionellosis severity may vary according to health status and could range from a mild febrile illness (called Pontiac fever) to a potentially fatal form of pneumonia (called Legionnaires’ disease, [LD]) [1]. LD case fatality rates ranges between 5–30% and up to 50% for individuals with compromised health status [2]. LD occurs as sporadic cases or as outbreaks, and is an important cause of both community and nosocomial-acquired pneumonia [1]. *Legionella* is found in natural and man-made aquatic environments, such as cooling towers and hot water plumbing infrastructures [2]. Water systems provide optimal growth conditions for *Lp* and help its transmission by generating aerosols [1].

*Legionella pneumophila* serogroup 1 (*Lp*1) is the most common etiological agent causing waterborne disease in the United States and Canada [3,4]. In the United States, the reported legionellosis incidence rates have increased almost 3-fold during 2000 to 2009 [5] and from 2001 to 2006, *Legionella* was identified to be the agent causing 29% of waterborne outbreaks [6]. In the province of Quebec, legionellosis has been a mandatory notifiable disease since 1987. Since the beginning of 2000s, about 10 outbreak investigations were conducted in the province, with the largest in 2012 in Quebec City where 182 cases were declared, including 13 fatalities [7]. This major outbreak led government authorities to implement a provincial registry for water cooling towers and also, legal obligations for owners to possess a maintenance plan and to perform monthly inspections and *Legionella* testing [8].

The microbiological aspect of an investigation is to seek evidence linking the source of the outbreak to the cases, by comparing *Legionella* isolates from environmental samples with those from patients. Accurate discrimination among *Legionella* isolates is important in order to identify cases with a common source of infection and the transmission routes of the microorganism. Many genotypic methods have been applied to the epidemiological typing of *Lp*. Pulsed-field gel electrophoresis (PFGE) and amplified fragment length polymorphism (AFLP) are two methods based on digestion of genomic DNA with restriction enzyme [9]. These two methods have been used both in outbreak investigation and for assessing genetic diversity of sporadic cases [10–14]. However, even though information provided is useful, their efficiency varies and most of them are not standardized [15]. Since the last decade, these methods have been replaced by Sequence-based typing (SBT). SBT is a rapid, highly discriminatory, and reproducible seven-gene molecular typing method that has now become an internationally recognized procedure for genotyping *Lp* isolates [16–18]. Recent studies performed on isolates recovered from different parts of the world suggested a group of sequence types (STs) being predominant in sporadic cases and outbreaks [19–21].

The Laboratoire de santé publique du Québec (LSPQ), the provincial reference laboratory, routinely performs culture detection, confirmation and strain typing of *Legionella*. In this study, we investigate the types and frequencies of *Lp* strains in the province of Quebec between 2005 and 2015 in order to identify dominant clusters, therefore contributing to the global knowledge of *Lp* STs dynamics.

## Materials and Methods

### Study area

The province of Quebec is compromise of 7,979,663 inhabitants according to the 2011 Canadian Census (www.statcan.gc.ca). Nearly 80% of the population lives along the shores of the St. Lawrence River. This area has a temperate continental climate with four successive seasons. This climate is characterized by warm and slightly humid summers and cold winters, with a temperature range of approximately 30°C (www.gouv.qc.ca).

### Case definitions

A confirmed case of legionellosis is defined as a compatible clinical illness with laboratory confirmation of infection by at least one of the following: a positive urine antigen test, a positive culture, a positive immunofluorescence antigen test from respiratory specimens, lung tissue, pleural fluid, a four-fold increase of immunofluorescence antibody; an IgG titre of ≥1:128 [22]. Case records were extracted on April 6^th^, 2016 from Quebec’s notifiable infectious diseases databank, implemented in 1990. Annual incidence rates were calculated using population censes data provided by the Institut de la statistique du Québec. Information about genus and species of *Legionella*, and serogroups are available since 2008. However, cases of *Legionella* sp. and *Lp* for which the information on serogroup was not provided were reassigned as *Lp*1 when the specimen used for laboratory confirmation was a urine sample (for detection of urinary antigen).

### Strains acquisition

In the province of Quebec, hospital microbiology laboratories are not required to send *Legionella* isolates to the LSPQ. Isolates from sporadic cases are typically sent to LSPQ for identification, confirmation or for further characterizations to the species or the serogroup levels. LSPQ is also the reference center for *Legionella* typing and receives isolates from patients and from the environment in outbreak contexts. Finally, environmental isolates from cooling towers, hospital water systems or private water systems are also sent to LSPQ for further identification and confirmation.

### Strains identification

At LSPQ, *Lp* isolates were confirmed by indirect fluorescent antibody (IFA) assay (Monofluo *L*. *pneumophila* kit, Biorad, Canada). Serogroup 1 (SG1) strains were ascertained by agglutination test slide (*L*. *pneumophila* antisera, Denka Seiken, Japan). *Lp* isolates other than SG1 and *Legionella* sp. other than *pneumophila* were sent to the National Microbiology Laboratory (NML) for identification by 16S rRNA sequencing and serogrouping.

### SBT typing

We conducted SBT analysis on a large set of *Lp* isolates to investigate the provincial STs distribution. This set was comprised of isolates thought to have caused outbreaks, based on epidemiological investigations (isolates from patients and epidemiologically linked environmental reservoirs) as well as isolates from sporadic cases or from the environment sent to LSPQ for reference testing. SBT was performed at LSPQ or at NML according to the European Working Group for *Legionella* infections (EWGLI) protocol [17,18,23]. Sequences obtained by Sanger sequencing were analyzed with BioNumerics software (Version 7.5, Applied Maths, Austin, TX, USA) and compared to the EWGLI database for assigning the ST. New allelic profiles were submitted to the EWGLI SBT-database (http://www.hpa-bioinformatics.org.uk/legionella/legionella_sbt/php/sbt_homepage.php).

### Phylogenetic and allelic diversity analysis

Multiple sequence alignments of concatenated DNA sequences were clustered with the unweighted pair group method with arithmetic mean (UPGMA) algorithm by using BioNumerics software. Cluster definition was fixed at ≥98% of similarity. Examination of the relationships between STs was conducted using the eBURST v3 website (http://eburst.mlst.net). Because we had a small dataset, the eBURST groups were obtained with a less stringent definition, in which STs are included within the same group if they share identical alleles at five or six of the seven SBT loci with at least one other ST. For the eBURST analysis of the data from the EWGLI database (data from 2016-04-13), we used a more stringent group definition, in which STs are included within the same group only if they share identical alleles at six of the seven SBT loci. In both cases, STs that cannot be assigned to any group are called singletons. With the eBURST algorithm, members of a group are all believed to be descended from the same founding genotype (the primary founder). The statistical confidences for the founders were assessed using 1000 bootstrap re-samplings [24].

## Results

### Evolution of the incidence rate

**Fig 1:**
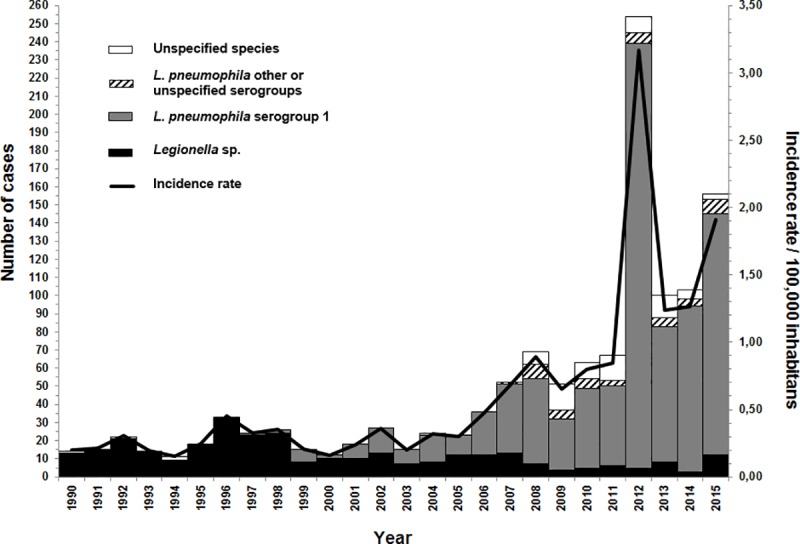
Number of cases per year, by species and serogroup, and annual incidence rate of legionellosis, province of Quebec, 1990–2015.

Fig 1 shows the number cases and annual incidence rates of legionellosis reported in the province of Quebec from 1990 to 2015. From 1990 to 2005, the incidence rate remained stable, with a mean of 0.27/100000 inhabitants. From 2006, incidence rates increased, reaching 1.91/100000 inhabitants in 2015 (excluding cases from the 2012 outbreak). The raise was particularly important after the 2012 outbreak. According to these data, the majority of cases was diagnosed by urinary antigen detection since its gradual introduction in 1998. The rate of diagnosed cases by urinary antigen detection was an average of 37% from 1998 to 2005 and an average of 77% from 2006 to 2015.

### Genotype diversity of isolates

**Fig 2:**
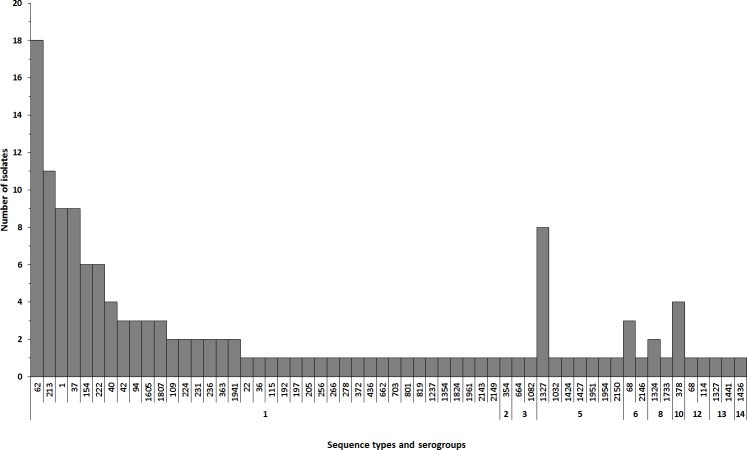
Sequence type (ST) distribution amongst legionellosis sporadic cases and their serogroup between 2005 and 2015 (n = 141). For ST-1327, 1 isolate has cross reaction with serogroup 5 and 9 antibodies. For ST-378, 1 isolate was from serogroup 10 and 3 has an inconclusive serogroup.

From 2005 to 2015, a mean of 25% of the annually reported case isolates were sent to LSPQ (range 12% to 36%). This represents a total of 213 isolates. Among these, 186 were *Lp* isolates and 181 were available for SBT typing (141 sporadic cases and 40 outbreak related cases). SBT data from 103 environmental isolates obtained from outbreak investigation or special typing requests were available from the same period of time. Fig 2 shows the distribution of the SBT types for the 141 human sporadic cases. SBT results indicated that 141 human sporadic cases belonged to 57 different STs. Of note, 11 STs were newly reported to the SBT database, 9 were new combination of previously known alleles and the remaining were STs with one new allele. Amongst the 108 sporadic cases of SG1 typed by SBT, ST-62 was the most frequent (16.6%), followed by ST-213 (10.2%), ST-1 (8.3%) and ST-37 (8.3%). Amongst other serogroups, ST-1327 (SG5) (27.3%) and ST-378 (SG10) (12.2%) were the most frequent.

Table 1 shows STs for outbreak associated to human and environmental isolates. Amongst the 10 outbreaks investigated by the Quebec’s public health authorities from 2005 to 2015, isolates from 8 outbreaks were available for typing. A different ST was involved for each outbreak investigated, except for two outbreaks where ST-62 was involved. Notably, all the community associated outbreaks, potentially or confirmed to be associated with a cooling tower were caused by *Lp*1 strains, by opposition to hospital associated outbreaks that were caused by serogroups of *Lp* other than SG1. Table 2 shows the STs for the remaining environmental isolates where ST-1 represents the more frequent (26.5%).

**Table 1:** *Legionella pneumophila* outbreaks investigated by LSPQ between 2005 and 2015.

Year	Location	Serogroup	Number of human isolates typed ^a^	Number of environnemental isolates typed ^b^	Sequence type
2008	Health care associated	SG5	4	0	ST-1327
2010	Community associated	SG1	3	0	ST-15
2011	Health care associated	SG10	2	8	ST-378
2012 ^c^	Community associated	SG1	22	2	ST-62
2013	Community associated	SG1	2	1	ST-256
2014	Community associated	SG1	2	0	ST-42
2015	Health care associated	SG5	2	9	ST-1427
2015	Community associated	SG1	3	0	ST-62

^a^ More clinical cases than human isolates typed could have been associated with an outbreak.

^b^ Only the isolates from the outbreak associated sequence type are shown.

^c^ For this outbreak, SBT typing was used as second typing method after pulsed-field gel electrophoresis typing. For the total number of isolate typed, see Lévesque *et al*. [7]

**Table 2:** *Legionella pneumophila* environmental isolates typed at LSPQ between 2005 and 2015.

Serogroup	Sequence type^b^	Number of isolates
1	**ST-1**	22
	ST-284	5
	**ST-36**	2
	ST-150	2
	ST-1940	1
	**ST-40**	1
	**ST-154**	1
2	ST-1354	10
	ST-1957	1
	ST-1958	1
5	**ST-1427**	3
	**ST-1327**	2
6	**ST-68**	9
	ST-1339	1
8	**ST-1324**	1
9/10 ^a^	**ST-378**	20
Inconclusive	ST-1956	1
Total		83

^a^ For ST-378, 5 isolates from serogroup 9 and 15 isolates from serogroup 10.

^b^ ST in bold are also associated with human isolates in this study.

### Relationships between isolates

The population structure of the 108 sporadic cases of SG1 typed by SBT was analysed using the concatenated sequences of the 7 loci. UPGMA algorithm was used to construct the dendrogram showed in S1 Fig. The UPGMA dendrogram contains 4 major clusters (A to D) of related STs. Cluster B contains the majority of the strains (n = 61) and the three most frequent STs in our database (ST-62, ST-213 and ST-1). Of note, the single isolate of ST-1354 clustered distantly from the 4 major clusters. This is because this strain possess a *neuAh* allele (*neuAh*-207), normally found in SG2 strains. This *neuAh* allele was previously identified in 4 other STs from *Lp*1 strains according to the EWGLI database.

**Fig 3:**
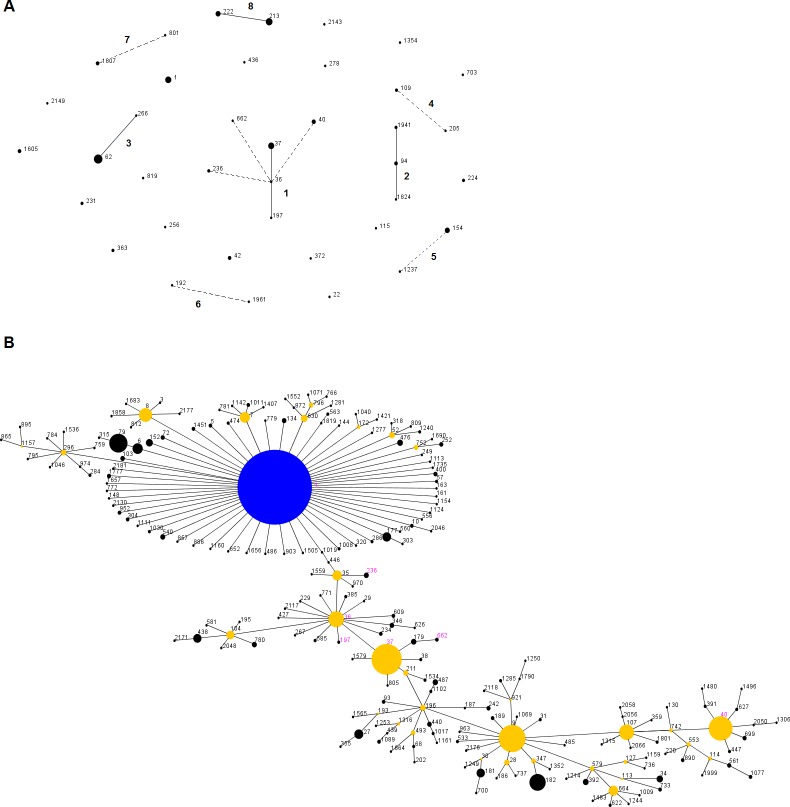
eBURST analysis of *Legionella pneumophila* SG1 sequence type. Panel A shows the 141 sporadic cases of our study. Full lines link single-locus variants and dashed lines double-locus variants. Group numbers are also indicated. Panel B shows the group 2 of the EWGLI-SBT database eBURST analysis from SG1 strains. Sequence types in pink are from our study. Blue dot represent the primary founder and yellow dots the subfounders. For both panels, the size of the dot is proportional to the number of isolates in the analysed collection.

The eBURST algorithm was used on the same dataset in order to identify the clonal lineages of our strains. Using the less stringent definition for our dataset alone, eBURST algorithm identified 8 groups and 17 singletons as shown in Fig 3A. The 8 groups include 21 STs (n = 76 strains). Groups created by the eBURST algorithm were congruent with the topology of the UPGMA dendrogram, where eBURST groups 2 and 7 were part of the UPGMA cluster A, groups 3, 4, 6 and 8 were part of the cluster B, and group 5 were part of the cluster D. Of note, eBURST group 1 was exclusively composed of the STs in the cluster C. We next did an eBURST comparative analysis of our dataset with the entire *Lp*1 strains available in the EWGLI database (n = 8525 strains, 1317 STs) using the more stringent definition. With this method, 61 groups were created. All but one (ST-1354 which is a singleton) of our strains clustered in a group. Fig 3B shows the group 2 that contains our strains from ST-36, ST-37, ST-40, ST-197, ST-236 and ST-662 (group 1 from Fig 3A, cluster C from S1 Fig), in addition with ST-1, which is the predicted founder of this group.

## Discussion

The real burden of legionellosis is greatly underestimated. Although comparison of surveillance data from different countries can be problematic considering differences between case definitions, type of surveillance system and method of diagnosis used, most countries have experienced increasing incidence rates for the last two decades [1,25]. This can be explained by several factors including: improved diagnosis, enhanced reporting and surveillance systems, increases in urinary antigen test use as well as an increasingly aging and immunocompromised population [1,26,27]. These factors can also apply to the two-fold incidence rate increase observed in Quebec from 2005 to 2011. The further increase of incidence rate observed after the Quebec City 2012 outbreak is possibly due in addition to enhanced physician awareness. The impact of the implementation of the 2014 water cooling towers maintenance plan in Quebec will certainly have an influence on incidence rate of legionellosis in the future.

The fact that all the hospital associated outbreaks were caused by serogroups of *Lp* other than SG1 is an interesting finding. Many other hospital associated cases or outbreak worldwide were previously associated with serogroups other than SG1 [10,28–32]. This may be explained in part by the fact that hospital water networks are often colonized by *Lp* strains other than SG1 [33–41]. A study performed in the province of Quebec in the 90s showed that more that 70% of the 84 hospital tested had their water distribution system contaminated with *Lp* strains other than SG1 [42].

The standardised SBT scheme for *Lp* presents an important advancement in the study of the molecular epidemiology of LD. It has been shown that the results obtained by different typing scheme for *Legionella*, such as PFGE and AFLP, are not necessarily concordant between them and also with SBT [13,14]. Implementation of SBT method has yielded comparable data worldwide and has been shown to be applicable in investigation of legionellosis outbreaks [1].The genetic variability of *Lp* has been assessed by SBT for countries such as the United-States, Canada, United-Kingdom, Germany, Spain, Belgium, Portugal, Italy, South Korea, China and Japan [3,19–21,43–49]. Despite the fact that each region harboured its own *Lp* genetic organisation, ST-1 is clearly the dominant genotype worldwide, both in clinical and environmental isolates. However, its virulence could be variable depending of its origin. In a large *Lp*1 strain characterisation study in United-States, ST-1 stains were by far the most common genotype associated with sporadic cases and were also recovered from water samples. However, this genotype was never associated with outbreaks originating from USA investigated by the Centers for Disease Control and Prevention [19]. Also in our study, ST-1 was never implicated in outbreaks investigated by LSPQ, even if this genotype is found frequently in the environment. This contrast also with our neighboring province, Ontario, where ST-1 has caused outbreaks [50]. However, by opposition to all other studies, ST-62 was the most frequently found genotype in Quebec in sporadic cases and it also caused two important outbreaks. This sequence type was found through the study period and was also found in many different regions of the province of Quebec. According to the EWGLI database, this ST represents 3.6% of the isolates typed and have been found in North America and Europe. In Canada, in addition to Quebec and Ontario, this sequence type was also recovered from a human case in Nova Scotia [3]. Despite the fact that ST-62 is rarely found in environmental samples in Quebec, compared with ST-1 strains, it seems to have a particular virulence potential. Further studies involving whole genome sequencing analysis and in vitro experiments will have to be conducted in order to verify this hypothesis.

*Legionella* population structure in Quebec showed significant genetic diversity as previously shown in studies from other parts of the world [3,19–21,43–49]. The fact that 4 major clusters have been identified amongst Quebec human isolates suggest that multiple *Lp* genotypes have evolved concomitantly. This finding is enhanced by the fact that eBURST analysis identified singletons by a majority, even using a less stringent definition. Our population structure is also in line with the worldwide population structure of *Lp* as showed by the eBURST analysis of the EWGLI SBT database. More recently, whole genome sequencing data were used to determine the *Legionella* population structure in comparison with SBT data [51,52]. The authors reported that in general, the SBT sequence type accurately reflects the whole genome based genotype. However, they found that strains from the same ST can evolve differently, reflecting the high level of recombination in *Lp*.

Our study has some limitations. First, like other countries, the majority of the diagnosis in Quebec is made by urine antigen detection. This implies that the strain types collected represent only the tip of the iceberg of the legionellosis burden and induces a selection bias towards SG1. This is also related to the fact that the hospital microbiology laboratories are not required to send *Legionella* isolates to the LSPQ. However, the strains typed in our study represent cases well distributed in the province of Quebec territory and all across the study period. Secondly, based on notifiable disease system data, we reassigned cases of *Legionella* sp. and *Lp* for which the information on serogroup were not provided but one of the specimen used for laboratory confirmation was a urine sample as *Lp*1. However, cross-reactions with other serogroups have been reported [53].

In conclusion, this study reports the molecular characterization of *Lp* strains during a 10 year period. We observed an important increase in the incidence rate of legionellosis in Quebec. The recent major Quebec City outbreak caused by *Lp*1 ST-62, and the fact that this genotype is the most common in the province support the ongoing whole genome sequencing characterization of this particular sequence type in order to understand its evolution and associated virulence factors.

## Supporting Information

S1 Fig Unweighted pair group method with arithmetic mean (UPGMA) dendrogram of the 108 *Legionella pneumophila* SG1 sporadic cases typed by SBT using the concatenated sequences of the 7 loci. Clusters are represented by letters. Scale represents the similarity percentage. ST = sequence type.
(TIF)
